# Sirenomelia

**Published:** 2010-08-14

**Authors:** Kanchan Kayastha

**Affiliations:** Department of Pediatric Surgery, The Children's Hospital and the Institute of Child Health Lahore, Pakistan

A baby was delivered to a 28-year old mother with fused limbs, no feet, absent external genitalia and imperforate anus.

The newborn at arrival in emergency room of our institution was hypothermic and cyanosed. Neonate was resuscitated. Clinical examination following resuscitation revealed fused lower segment of the body below pelvis into a single limb with no feet. Posture of the lower torso was that of alphabetic letter L when viewed from the back. There were no openings for urogenital system and anal opening was also absent (Fig. 1).

**Figure:**
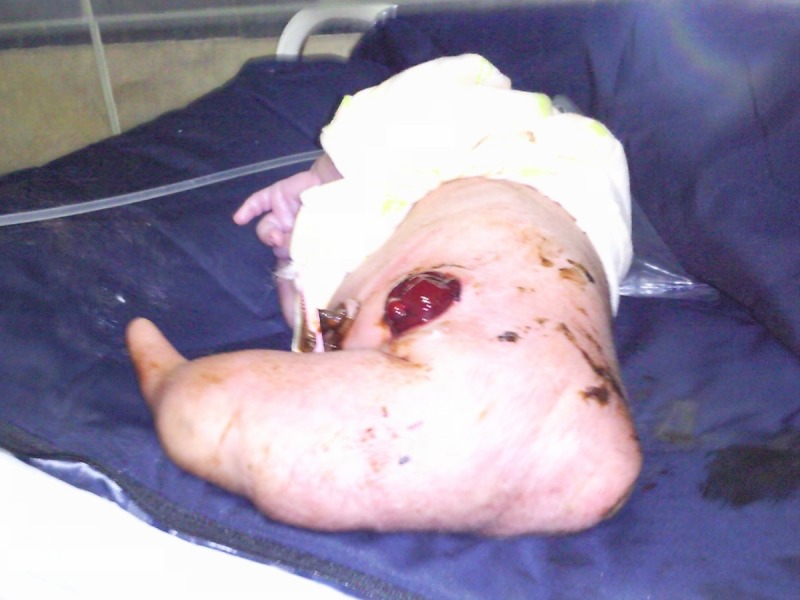
Figure 1: Patient with imperforate anus and absent external genitalia. Lower limbs fused into a single limb

The ultrasound of the patient revealed bilateral renal agenesis, and the gonads and urinary bladder were also not visualized. A sigmoid loop colostomy was formed for imperforate anus. The clinical condition of the baby deteriorated afterwards and expired at the end of first day of life.

## DISCUSSION

Sirenomelia is a rare congenital anomaly characterized by partial or complete fusion of lower limbs and usually associated with other severe anomalies. It is considered, by many authors, as severe form of caudal regression syndrome. The associated anomalies may include bilateral renal agenesis, complete or partial agenesis of genitourinary system, imperforate anus, absence or ambiguous external genitalia, single umbilical artery, lung hypoplasia and vertebral and cardiac anomalies [1 , 2]. In our case the associated anomalies were complete agenesis of urogenital system and imperforate anus.

Etiology of sirenomelia is uncertain and various theories have been proposed to explain its origin. An embryonic insult to caudal mesoderm between 28-32 days of gestation and vascular hypo-perfusion (arterial steal) has been proposed as possible factors. Others associated the condition with maternal diabetes mellitus, exposure to teratogens and genetic predisposition [3].

Depending upon the degree of fusion of lower limbs and feet, sirenomelia can be classified into three forms

Simpus apus (no feet, one tibia, one femur)
Simpus unipus (one foot, two tibia, two fibula, two femur)
Simpus dipus (two feet, two fused legs) flipper like popularly known as mermaid [4].

In our case the exact type of the anomaly cannot be proposed in absence of radiographic findings, however, clinically the baby can be placed in simpus apus group as the patient did not have feet. In expert hands the antenatal sonographic diagnosis of sirenomelia can be made as early as 18 weeks of gestation and a better option of termination of pregnancy can be advised in order to avoid physical and psychological stress to parents and the family.

## Footnotes

**Source of Support:** Nil

**Conflict of Interest:** None declared
